# Three Types of Beetroot Products Enriched with Lactic Acid Bacteria

**DOI:** 10.3390/foods9060786

**Published:** 2020-06-14

**Authors:** Vasilica Barbu, Mihaela Cotârleț, Carmen Alina Bolea, Alina Cantaragiu, Doina Georgeta Andronoiu, Gabriela Elena Bahrim, Elena Enachi

**Affiliations:** 1 Faculty of Food Science and Engineering, Dunărea de Jos University of Galati, 111 Domneasca Street, 800201 Galati, Romania; vasilica.barbu@ugal.ro (V.B.); mihaela.cotarlet@ugal.ro (M.C.); carmen.bolea@ugal.ro (C.A.B.); georgeta.andronoiu@ugal.ro (D.G.A.); gabriela.bahrim@ugal.ro (G.E.B.); 2 Research and Development Center for Thermoset Matrix Composites, Dunărea de Jos University of Galati, 111 Domneasca Street, 800201 Galati, Romania; alina.cantaragiu@ugal.ro

**Keywords:** beetroot, lactic acid bacteria, betalains, functional food products

## Abstract

Beetroot (*Beta vulgaris* L.) represents a very rich source of bioactive compounds such as phenolic compounds and carotenoids, among which the most important being betalains, mainly betacyanins and betaxanthins. The beetroot matrix was used in a fresh or dried form or as lyophilized powder. A 10^12^ CFU/g inoculum of *Lactobacillus plantarum* MIUG BL3 culture was sprayed on the vegetal tissue. The lactic acid bacteria (LAB) viability for all the products was evaluated over 21 days, by microbiological culture methods. The antioxidant activity of the obtained food products was correlated to the betalains content and the viability of LAB. The content of polyphenolic compounds varied between 225.7 and 1314.7 mg L^−1^, hence revealing a high content of bioactive compounds. Through the confocal laser scanning microscopy analysis, a large number of viable probiotic cells were observed in all the variants but especially in the fresh red beet cubes. After 21 days of refrigeration, the high content of *Lb. plantarum* (CFU per gram) of the food products was attributed to the biocompounds and the nutrients of the vegetal matrix that somehow protected the bacterial cells, and thus maintained their viability. The obtained food products enriched with probiotic LAB can be regarded as new functional food products due to the beneficial properties they possessed throughout the undertaken experiments.

## 1. Introduction

Beetroot (or red beet) is an edible taproot of *Beta vulgaris* L. subsp. *vulgaris* (var. *conditiva*) species that is phylogenetic framed in the *Betoideae* subfamily, in the *Amaranthaceae* family and is the most important crop of the large order *Caryophyllales*. The specie has many varieties that are grown throughout the Americas, Europe, and Asia. Beetroot, through its rich content in betalains, phenolic compounds, and carotenoids, is an important source of biocompounds with antioxidant properties and may represent a strong alternative to synthetic dyes. Natural pigments without any toxic effect are extremely valuable and they have important applications in the field of medicine, food, cosmetic or pharmaceutical industries. Betalains are a group of secondary plant phenolic metabolites, being water-soluble vacuolar chromoalkaloids found in many plants with health benefits for humans, especially regarding their antioxidant, anti-inflammatory, antiviral, even anti-tumoral activities [1,2,3]. Tesoriere et al. [4] and Reddy et al. [5] also proved the betalains role concerning the inhibition of lipid peroxidation, a very important process in the cholesterol metabolism. The main betalains that are found in beetroot are the red-violet betacyanins and yellow-orange betaxanthins [6,7] and, so far, several other natural derivatives have been described [8,9,10]. Among betacyanins, betanin, known as CI Natural Red 33 or E162, is the only betalain approved to be used in food, being almost totally extracted from beetroot crops [11] whereas among betaxanthins, indicaxanthin is the most important. Apart from betalains, other types of phenolic compounds have been identified such as small amounts of gallic, syringic, and caffeic acids and flavonoids [12]. Therefore, beetroot extract has shown a strong phytochemicals pattern which resulted in an increased researchers’ interest in the last decades. For these reasons, red beet represents a rich source of bioactive compounds and could be used to develop functional foods [1,6].

Lactic acid bacteria (LAB), especially Lactobacilli and their metabolites play a key role in improving the microbiological quality and also in increasing the shelf life period of many functional, fermented food products. Lately, the biotechnological studies in the food industry domain have designed several commercial products that contained a single probiotic starter strain or a bacterial consortium. Furthermore, during recent years, it was thoroughly proved that the regular consumption of viable probiotics can determine a number of health benefits such as: a decreasing cholesterol level [13,14], anti-diabetic effect [15,16,17], alleviating diarrhea and constipation, improving the lactose intolerance and the human gut microbiome, in general [18,19], strengthening the immune system [20] or even antitumoral effects [21,22].

This study followed the design of some new functional food products by combining the red beetroot under different forms with LAB, with a strong focus on the beneficial effects for health. The main objectives of the present research were to analyze these original products from the microbiological, biochemical, textural, ultrastructural point of view in order to highlight their functionality. The CIE color parameters as well as digestibility were also studied in order to further highlight the nutritional value of the newly designed products.

## 2. Materials and Methods

### 2.1. Lactic Acid Bacteria Strain

The *Lactobacillus plantarum* BL3 strain was used for this analysis from the Microorganisms Collection of the Bioaliment Research Platform (acronym MIUG) at −70 °C in MRS broth (De Mann, Rogosa and Sharpe—Sigma Aldrich, Darmstadt, Germany) supplemented with 20% (*v*/*v*) glycerol. The primary culture of lactic acid bacteria (LAB) was obtained after its cultivation on MRS broth (for 12 h at 37 °C) in order to obtain the mid-logarithmic phase cultures that were further used in our experiments. The cells were harvested by centrifugation at 4800 rpm min^–1^, at 4 °C, for 10 min and were washed twice with sterile 0.85% saline solution. A 10^12^ CFU mL^−1^ inoculum of *Lactobacillus plantarum* MIUG BL3 was used to spray the vegetal tissue.

### 2.2. Sample Preparation

The beetroot that was used for all the experiments was purchased from the local market. The beetroot was washed, peeled, and cut under aseptic conditions by using a food processor into cubes (with an edge of 5 mm ± 0.2 length), as slices or as thin chips (with an area of 2–3 cm^2^). The vegetal matrix was split into equal amounts of 100 g each, transferred to sterile Petri dishes and exposed at the UV light, for 30 min, by using a SafeFastElite 215S Microbiological Safety Cabinet (Faster, Cornaredo, Italy). On the fresh beetroot cubes, the bacterial suspension was sprayed in a 5:1 (*w*/*v*) ratio (F variant). After that, half of these samples (F) were packed into sterile, single use plastic zip-lock bags and refrigerated at 4 °C for the microbiological analysis in order to determine the shelf life. The other half of samples were frozen at −80 °C (Angelantoni Platinum 500+, ALS, Massa Martana, Italy) and then freeze-dried at 10 mBar and −50 °C, for 48 h, using a CHRIST ALPHA 1-4 LD plus equipment (Martin Christ, GmbH, Osterode am Harz, Germany) until constant weight was obtained (with a 4.25 ± 0.2% water content). The freeze-dried samples were then ground, portioned, and packed identically in quantities of 10 g powder (FDP) per sachet and were kept at the room temperature.

The chips were slowly dried (at 42 °C for 12 h) until a 12.55 ± 0.2% moisture content was reached, by using a cabinet Stericell 111 air dryer (Medcenter GmbH, München, Germany). On the dried chips, the LAB suspension (*Lactobacillus plantarum* BL3) was sprayed in a ratio of 3:1 (*w*/*v*), hence obtaining the dried chips (DC) variant. These samples (DC) were transferred into sterile, single use plastic zip-lock bags that were kept at room temperature in order to perform the microbiological analysis.

Thus, all three variants: fresh (F), dried chips (DC), and freeze-dried powder (FDP) have LAB in their contents. In the DC variant, LAB were sprayed after drying the chips, in the FDP variant, LAB were sprayed before lyophilization and grinding, and in the fresh variant (F), LAB were sprayed on the beetroot cubes and kept in this form at 4 °C.

### 2.3. Lactic Acid Bacteria (LAB) Viability

In order to estimate the shelf life period, the viability of the LAB in all the variants was weekly evaluated, by cultural methods, for a period of 21 days. The samples were homogenized in a 0.85% sterile saline solution using a Pulsifier equipment (Microgen Bioproduct, London, UK) at medium speed and maintained for 5 min. The homogenized samples were serially diluted with 0.85% sterile saline solution and spread over the MRS agar supplemented with 2% CaCO_3_. The plates were incubated at 37 °C for 48 h and the colonies were counted. The experiment was conducted in triplicate. The viability of *Lb. plantarum* BL3 was expressed as the log_10_ of the mean number of the colony forming units (that were counted for each dilution in three different plates) (CFU g^–1^).

### 2.4. Scanning Electron Microscopy (SEM)

The samples’ ultrastructures enriched with LAB were analyzed by scanning electron microscopy. The samples were attached on the aluminum stubs with double adhesive carbon conductive tape (12 mm W × 5 mL) and gold coated with 5 nm as thickness in an argon atmosphere by using SPI Supplies (USA) sputter coater. The surface micrographs of all the samples were obtained using the FEI Quanta 200 SEM (Fei Europe B.v. Eindhoven, The Netherlands) with a plasma current intensity of 18 mA, a pressure of 6 mBar, and a spot size of 10mm as working distance. The SEM images were taken at different magnifications between 100× and 5000×.

### 2.5. Confocal Laser Scanning Microscopy

The confocal images of the functional food products based on beetroot and LAB, were acquired with a Zeiss confocal laser scanning system (LSM 710) equipped with a diode laser (405 nm), Ar-laser (458, 488, 514 nm), DPSS laser (diode pumped solid state—561 nm) and HeNe-laser (633 nm). In order to observe in detail the vegetal microstructures and the *Lb. plantarum* BL3 cells, the Live/Dead Backlight bacterial viability stain kit (Molecular Probes, Eugene, OR, USA) was used according to the manufacturer’s instructions so that one drop was applied directly to the surface of each sample. It consisted of a two nucleic acid-binding stains mixture: SYTO9 which stained all the viable bacteria (shown in green), while the propidium iodide stained the non-viable bacteria (shown in red), after 15 min of dark incubation [23]. The excitation and emission wavelengths were 480 and 500 nm for SYTO9 and 490 and 635 nm for propidium iodide, respectively. The samples were observed with a Zeiss Axio Observer Z1 inverted microscope equipped with a 40× apochromat objective (numerical aperture 1.4). The 3D images were rendered and analyzed by a ZEN 2012 SP1 Black edition software. A minimum of twenty fields were evaluated, all the viability counts being determined in two independent experiments, with each assay being performed in triplicate.

### 2.6. Hardness Measurement

The texture of the beetroot dried chips (including hardness, porosity, crispness, chewiness, springiness) was determined using a Brookfield CT3-1000 texture analyzer (Ametek Brookfield, Middleborough, MA, USA), equipped with a 4 mm diameter steel cylinder (TA44). The stress at the maximum force was evaluated using a puncture test in the center of each dried chips. The samples had 30 mm length, 20 mm width, and 3 mm depth. The Texture Profile Analysis method (TPA) was used to determine the hardness and springiness of the samples. The stress at the maximum force was related to the hardness of the beetroot chips. The test speed was set at 1 mm s^–1^, with a target distance of 2 mm and a trigger load of 0.067 N. Before each experiment, a randomly chosen single beetroot chip was placed on the bottom parallel plate and compressed. The compression experiments were performed in 15 replications. The average force and energy required to cause deformation were determined on the basis of force-deformation curves. The beetroot chips were analyzed weekly, for a period of 21 days.

### 2.7. Betalains Quantification

In order to extract the pigments from the plant tissue, a weak acid solution (0.5% citric acid and 0.1% ascorbic acid) was used in accordance to Neagu and Barbu [24] so that the maximum content was obtained with the minimum pigment degradation. The liquid/solid ratio used in this study was 5:1 (*w*/*v*) and the mixture was homogenized for 30 min by magnetic stirring and afterwards filtered. The operation was repeated three times and the total extract was evaporated under mild conditions under vacuum at a temperature that did not exceed 40 °C with a Christ RVC 2-18 equipment (Martin Christ, Germany) until the aqueous extract was concentrated. The betalains content (B) of the extracts were determined spectrophotometrically at a wavelength corresponding to the maximum absorption of each of the betalains with a JENWAY 6505 UV/Vis equipment, according to the Lambert Beer’s law: A = log(I_10_/I) = ε × L × c. The following formula was further used: B (mg/L) = [(A_i_ × F_d_ × MW × 1000)/(ε × l)], where A_i_ is the absorption at 538 for betacyanins and 480 nm for betaxanthins, F_d_ is the dilution factor and l the path length of the cuvette (1 cm). To quantify the betacyanins (BC) and betaxanthins (BX), the molecular weights (MW) and molar extinction coefficients (ε) of the representative compounds were undertaken i.e., betacyanins (ε = 60,000 L mol cm^−1^ in H_2_O; MW = 550 g/mol) and betaxanthins (ε = 48,000 L/mol cm in H_2_O; MW = 339 g/mol). The betalains content was assessed with the formula (B) (mg/L) = BC + BX [6,25,26]. Fresh red beet cubes without LAB were analyzed as the control sample.

### 2.8. Color Measurement

In order to assess the color difference between the samples and also the Hunter color L*, a*, and b* parameters a CR-410 Chroma Meter (Konica Minolta Sensing Americas, Ramsey, NJ, USA) was used. The values were determined after the samples were ground into a fine powder. The information given by the L*, a* and b* parameters was generally expressed as the total color of the beetroot samples, the positive L* values representing the brightness and negative values representing lusterless or dullness, a* for the redness (+) to greenness (−), and b* for the yellowness (+) to blueness (−). The second method for the browning assessment was assessed by using the following formula: Browning Index (BI) = (100 (x − 0.31))/0.17, where x = (a* + 1.75L*)/(5.645L* + a* − 0.3012b*), according to Chandran et al. [27].

### 2.9. Antioxidant Activity

The antioxidant capacity was determined as described by Yuan et al. [28], by using a DPPH (2,2-diphenyl-1-picrylhydrazyl, Fluka Chemie, Fluka Chemie GmbH, Buchs, Switzerland) methanolic solution (0.1 M) with 30 min as the reaction time, and quantified using a Trolox (6-hydroxy-2,5,7,8-tetramethylchroman-2-carboxylic acid) calibration curve, under the same conditions. The antioxidant activity of the beetroot extract was measured by a spectrophotometric method at 517 nm. The percent inhibition of DPPH was calculated as follows: Antioxidant activity (%) = ((Absorbance of the blank−Absorbance of the sample)/Absorbance of blank) × 100. Fresh red beet cubes without LAB were analyzed as the control sample.

### 2.10. Total Phenolic Content

The total phenolic content (TPC) was determined using the Folin-Ciocalteu reagent and gallic acid as a standard after the method of Turturică et al. [29]. The beetroot extracts were totally dissolved in ddH_2_O (1 mg/mL). Shortly after, 200 μL aliquots of the resulting solution were mixed with 125 μL of 2 N Folin–Ciocalteau’s phenol reagent, diluted 1:2 (*v*/*v*). After 3 min of mixing, 125 μL of 20% Na_2_CO_3_ and 550 μL of deionized water were added. The resulting mixture was kept for 30 min in the dark, at room temperature; after that the mixture was centrifuged at 8200× *g* for 10 min. The absorbance was measured at 765 nm. The results were expressed as mg of gallic acid equivalents (GAE) per liter. Fresh red beet cubes without LAB were analyzed as the control sample. Each sample was measured in triplicate.

### 2.11. Digestibility

To achieve the in vitro digestibility of the obtained products, a static method was used as stated by Croitoru et al. [30], with small modifications. The products were shredded and afterwards mixed with a 10 mM Tris-HCl, pH 7.7, at a ratio of 1g of product to 10 mL of buffer solution. In order to thoroughly simulate the digestive conditions, a gastric mixture containing 20 mg of pork pepsin and 20 mL of HCl 0.1 N to reach the pH 2.0. The samples were further incubated at 37 °C on an orbital shaker (Optic Ivymen System, Grupo-Selecta, Barcelona, Spain) at 170 rpm. Regarding the intestinal digestibility, the mixture contained 40 mg of pancreatic enzymes and 20 mL of sodium bicarbonate 1M, the mixture having a pH of 7.7. The determination of betalains content was assessed as described previously.

### 2.12. Statistical Analysis of Data

Unless otherwise stated, the data reported in this study represent the averages of triplicate analysis and were reported as mean ± standard deviation. The analysis of variance (ANOVA) (*p* < 0.05) was carried out to assess the significant differences between values.

## 3. Results and Discussion

### 3.1. LAB Viability

Tripathi and Giri [31] stated that many factors were found to influence the viability of probiotic microorganisms in food products during production, processing, and storage such as fermentation and storage conditions (temperature, pH, medium, oxygen, etc.) protective agents, microencapsulation methods, food ingredients, processing operations (drying, freeze-drying etc.) [31].

**Figure 1:**
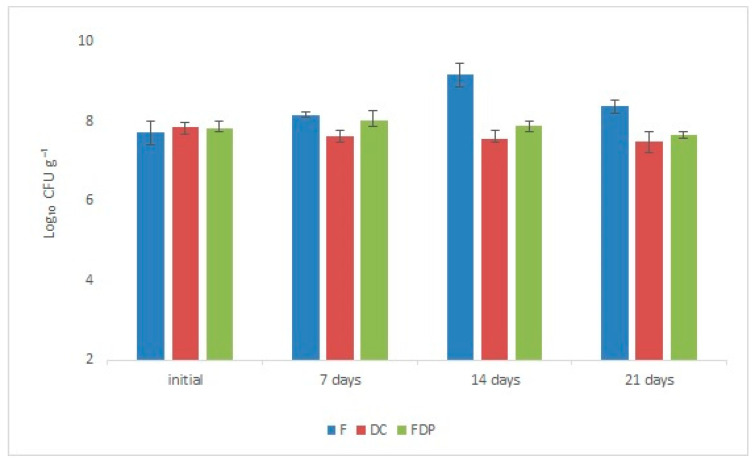
Viability of *Lactobacillus plantarum* BL3 in the experimental variants during 21 days of storage: F—Fresh beetroot cubes, DC—dried chips of red beet, FDP—freeze dried powder (data are the means ± SD).

The comparison survival of LAB between all the obtained products indicated a different viability behavior which in term depended on the processing method. The initial LAB content in all the experimental variants was evaluated immediately after the sample’s preparation. The content presented values between 5.14 × 10^7^ CFU g^−1^ (in fresh cubes of beetroot) and 7.05 × 10^7^ CFU g^−1^ (in dried beetroot chips). The higher value in the DC variant was at this rate, due to the fact that LAB suspension was sprayed, in a ratio of 3:1 (*w*/*v*), after the drying process. Although, at the beginning of the experiment, the LAB content started from a larger value in the DC version, the microbiological analysis showed that during storage, at room temperature, the number of probiotic lactic bacteria was slightly reduced, although the content was rather constant being still at a large magnitude order (10^7^ CFU g^−1^) even after three weeks (Figure 1). Surprisingly, the evolution of the *Lb. plantarum* BL3 strain in the fresh variant, registered a 100-fold increase from 5.14 × 10^7^ CFU g^−1^ to 1.46 × 10^9^ CFU g^−1^ (*p* ≤ 0.05), during the storage period at 4 °C, value attributed to the biochemical environmental conditions that were favorable for development and multiplication. Similar results were obtained also in the case of a patented product based on carrot slices fermented with *Lactobacillus strain* NCIMB 40,450 in which, after 28 days of fermentation, a concentration of 1–6 × 10^8^ CFU g^−1^ was reached [32] (EU patent no: 0536851). Regarding our results, in the fresh vegetal matrix, the tissue nutrients (carbohydrates and proteins as a source of C and N, respectively) are more accessible for the lactic acid bacteria, which explains the increase of the LAB number in the F variant. Because the accessibility of the nutrients was higher, the lactic fermentation process took place, which contributed to the special organoleptic impression of this functional product variant. The fresh product had a sourer taste and a softer texture compared to the other samples. It should be also emphasized that the lactic microbiota has not dropped below 10^6^ CFU g^−1^ in any of the proposed functional food products, even after three weeks, which allowed us to appreciate the shelf life of these products. The maintenance of a sufficiently large number of CFU per gram of product in the DC and FDP variants could be explained by the fact that the biocompounds and the nutrients of the vegetal matrix microencapsulated and protected the bacterial cells, and thus maintained their viability. The freeze-dried supplementary products better preserved the morphology of the probiotic microcapsules [31]. Additionally, the presence of yeasts, molds, or other mesophilic aerobic bacteria throughout the entire storage period has not been reported, probably because the lactic acid bacteria had an antimicrobial effect that inhibited the alteration microbiota, making these valuable products safe for consumption.

### 3.2. Scanning Electron Microscopy (SEM)

**Figure 2:**
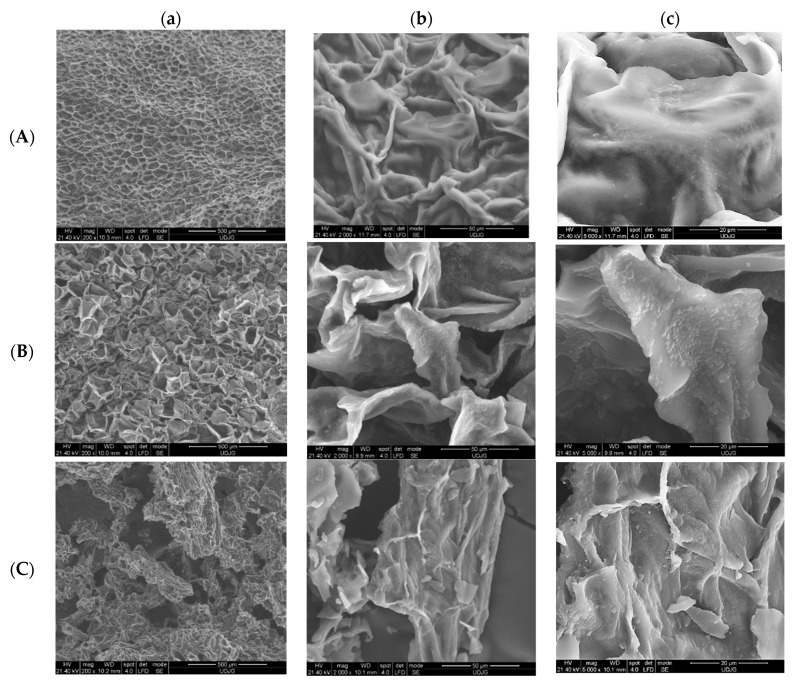
SEM images of the samples’ ultrastructure: (**A**)—fresh cubes with lactic acid bacteria (LAB); (**B**)—dried chips with LAB; (**C**)—freeze-dried powder with LAB; (**a**)—200×, (**b**)—2000×, (**c**)—5000×.

The scanning electron microscopy images showed the vegetal tissue with its isodiametric parenchymal cells and their whole cell walls (in fresh beetroot cubes—Figure 2A,a). The drying process at mild temperatures (42 °C) resulted in the changing of the cells shape, loss of turgor (Figure 2B,a), these modifications being more evidenced in the freeze-dried powder (Figure 2C,a) where the cells were smaller, at the same magnification (200×).

Much more interesting than the microscopic appearance of the plant tissue subjected to mild processing techniques was the appearance of the *Lb. plantarum* BL3 bacterial biofilm that adhered to the large surfaces of the plant cells walls (Figure 2b,c). The main characteristic of the biofilms was the formation of an extracellular polysaccharide (EPS) matrix, which provided protection of biocompounds and helped to create a microenvironment for the metabolic interaction of the population [33,34]. These extracellular capsules could be clearly observed for all the experimental variants (Figure 2A–C), especially at 5000× magnification (Figure 2c).

The biofilm was at its third stage after seven days after the inoculation and was characterized by the aggregation of cells into microcolonies that resulted from the simultaneous division and growth of the microorganisms. EPS helped to strengthen the bond between the bacteria and the substratum and stabilized the colony against any environmental stress. Thus, it could be observed that the amount of exopolysaccharides was even greater and the microcolonies were becoming more extensive as the processing technology was stronger (Figure 2c comparing between A, B, C).

### 3.3. Confocal Laser Scanning Microscopy

**Figure 3:**
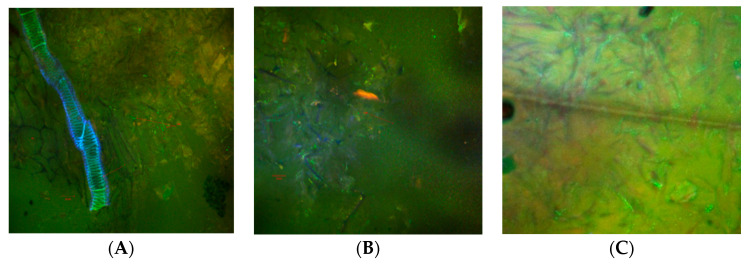
CLSM images of samples microstructure: (**A**)—fresh cubes with LAB; (**B**)—dried chips with LAB; (**C**)—freeze dried powder with LAB (*Lb. plantarum* BL3 viable cells—in green and the non-viable bacteria in red).

Confocal laser scanning microscopy images showed a huge number of viable LAB (colored in light-green) in all the variants but especially in the fresh red beet cubes (Figure 3A), where a significant multiplication of *Lb. plantarum* BL3 cells were observed during the refrigeration period, which was probably due to the rich environment offered by the beetroot sap. Moreover, the high moisture, high content of minerals, vitamins, biocompounds, and carbohydrates provided a supportive environment for the multiplication of *Lb plantarum* BL3 bacteria. About 80–90% of the *Lb plantarum* BL3 cells were viable in the dried chips (B) or in the freeze-dried powder (C), being observable by using the fluorescent dyes included in the LIVE/DEAD BacLight kit. These results were well correlated to those obtained by Moreno et al. [23] who assessed the LAB quantitative evaluation by indirect cultural methods. Additionally, our results are in accordance with the US FDA recommendations that state that the minimum probiotic count in a probiotic food should be at least 10^6^ CFU mL^–1^. Depending on the ingested amount (100 g in F and DC variants and 10 g in FDP supplement) and by also taking into account the effect of storage on the probiotic viability, a daily intake of 10^8^–10^9^ probiotic microorganisms will ensure the achievement of a probiotic action upon the human organism [31].

### 3.4. Measurement of Hardness

The results of TPA are presented in Table 1. The data represented the mean of three determinations. The hardness registered a slight increase during storage, from 0.73 N to 0.77 N, maybe due to the water loss. However, these values indicated a soft texture of chips, explained by the fact that during the dehydration process the equilibrum humidity was not achieved. At the same time, springiness declined from 1.57 mm to 1.52 mm, so that during storage the samples lost their elasticity. The texture parameters of beetroot chips did not vary significantly, asuring a constant texture quality during storage. Other characteristics correlated with the springiness were: gumminess index: 0.51 N, chewiness index: 0.41 N, similar to those obtained by Cui et al. [35] on apple chips.

### 3.5. Betalains Quantification, Total Polyphenols Content, and Antioxidant Activity

**Figure 4:**
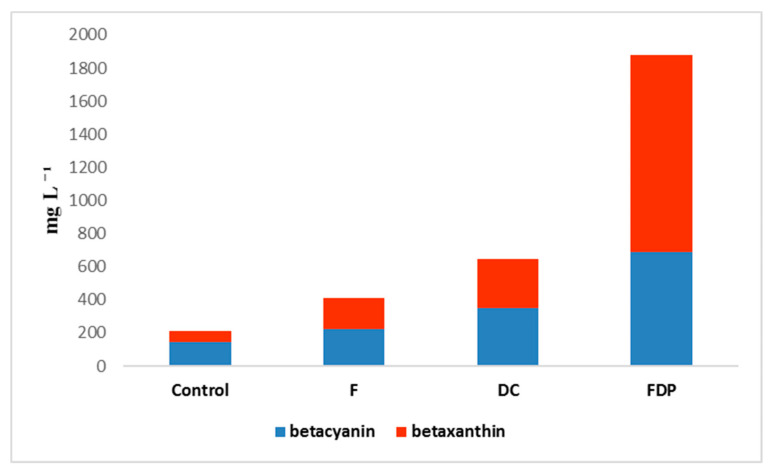
The betacyanin (BC) and betaxanthin (BX) content and their ratio in the experimental variants.

The content of betacyanin (BC) and betaxanthin (BX) in the fresh beetroot cubes without LAB (control samples) was 147.90 ± 5.204 mg L^−1^ and 64.68 ± 1.004 mg L^−1^, respectively (Table 2). The betacyanins represented 69.5% of the total betalalains. The DC and the FDP variant registered significantly higher BC and BX content compared to the F variant, respectively. For the DC variant, the BC and the BX contents were 349.25 ± 1.082 mg L^−1^ and 298.38 ± 5.854 mg L^−1^ whereas the FDP variant had a BC content of 689.79 ± 4.321 mg L^−1^ and a BX content of 786.69 ± 5.625 mg L^−1^. The addition of the selected LAB with probiotic effect caused a significant increase of the betalains content probably due to the increased extractability of these pigments. Betalains highest content was recorded in the FDP samples, the increase being more obvious in the betaxanthin case, and as such in the FDP variant the proportion of the two betalains classes was inverted (Figure 4). It is known that betaxanthins are more stable than betacyanins under the temperature and freeze-drying conditions [27].

The betalains content varies very much depending on the harvesting time, climatic conditions, variety, plant parts, maturation stage and extraction, or preservation methods [25,36]. Bucur et al. [25] showed that spring varieties had a much higher betalains content (almost double) compared to autumn red beet varieties due to the high temperature of the soil during the summer which in term caused the degradation by isomerization, decarboxylation, or by hydrolysis of the betalain molecule to betalamic acid [25,37,38]. The temperature and pH of the extraction medium and solid: liquid ratio are also very important factors that may modify the betalains values [24,36,39]. Freezing preservation has also been shown to halve the content of betalains in red beets [25]. Our results were similar to those obtained by Slavov et al. [40] when the whole beetroot was used as substrate.

When it comes to beetroot, the antioxidant activity was usually correlated with the betalains content [25]. Moreover, it is known that processed fruits and vegetables have lower antioxidant activity compared to the raw or fresh due to vitamin C degradation during processing [38]. Extremely variable values of antioxidant activity were also obtained by Kushwaha et al. [36]. The parameter depends on many factors related to variety or extraction methods, but the highest antioxidant (86.34%) activity was obtained at 50 °C and pH 3.5 [36]. Our functional food products registered a slight increase of the antioxidant activity compared to control samples, with the FDP displaying the highest values, 56.85% (Table 2).

The content of polyphenolic compounds varied from 225.7 to 1214.7 mg L^−1^ (Table 2), which was rather similar compared to the findings of Wruss et al. [7]. Kushwaha et al. [36] observed that with the increase of the extraction temperature from 40 to 55 °C, a slight increase of the phenolic extraction rate was also observed which might be due to the softening of tissue responsible for the weakening of the phenol–protein and phenol polysaccharide interaction in the plant tissue. The drying temperature of 42 °C favored the development of the lactic microbiota as well as the extraction of betalains and polyphenols.

### 3.6. Measurement of Color

The food quality stability requires a better understanding of the parameters that may influence it. The color plays an important role in the visual recognition and assessment of the surfaces and it has a great influence on the appearance and acceptance of food products. The beet root powder enriched with LAB, besides the intake of probiotics, could be used to improve the red color of dressings, mousses, jams, soups, jellies, ice creams, sweets, or breakfast cereals [27,41]. There are different modalities, arrangements, or formulas to appreciate the color of red beet by using L, a, and b CIE color parameters. Hue value was calculated as the angle that had the b/a tangent, such as the values from 0° and 90° corresponded to the hue that varied from pure red to pure yellow while the hue values between 90° and 180° corresponded to the hue that varied from pure yellow to pure green. From this perspective, the lowest hue value was determined in the case of fresh red beet cubes enriched with LAB (Table 3). In this variant, the red color was the most stabile probably because of the lactic fermentation produced by the *Lb. plantarum* BL3. This also correlated with the increased value of parameter a (Table 3). The fermentation in the fresh cubes variant (F) determined an increased lightness and red chromaticity, when compared to dried chips. The long drying, even at gentle temperatures, clearly affected the color of the functional product in our study. As mentioned by other researchers [27], the Hunter a/b ratio was found to be the best parameter to express the color degradation as quantitatively and correctly as possible [27]. Moreover, these authors showed that the color degradation measured as Hunter ‘a/b’ value followed a first order kinetics, where the rate constant increased with the increasing of temperature [27]. The a/b ratio of the chips obtained after 12 h of drying at 42 °C was 2.7 (Table 3), a result that was similar to those obtained by Chandran et al. [27] after 60 min of drying at 90 °C. It was also reported that the yellow pigments of beet root, betaxanthins, were more stable than the betacyanins (red pigments), although the degradation of both pigments was proportional [27,42]. The FDP variant showed intermediate values of all the color parameters compared to the F and DC variants, however closer to those of the fresh variant. Thus, it can be stated that drying, even at mild temperatures (42 °C for 12 h), affects more the CIE color parameters compared to the freeze-drying method. As a result, the FDP powder proved to possess ideal properties to be regarded as a food additive.

### 3.7. Digestibility of the Beetroot Products

**Figure 5:**
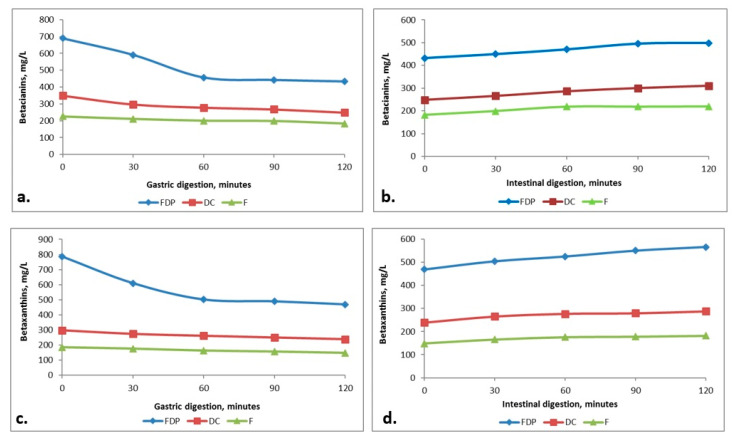
The digestibility of the beetroot products betacyanins (BC) and betaxanthins (BX) content during the gastric digestion (**a**,**c**) and the intestinal digestion (**b**,**d**).

The in vitro digestibility of the beetroot products has sought to assess the betalains behavior in both the gastric and intestinal juices. Prior to the absorption, the betalains could be hydrolysed in the gastrointestinal tract due to the environmental impact or due to some enzymes that are able to hydrolyze the betacyanins and betaxanthins. Regarding the other phytochemicals, previous studies have indicated that the conditions in the digestive tract (temperature, pH) and the β-glucosidases of the microflora could cause the hydrolysis of several important bonds. Another important factor may be the activity of the cytosolic β-glycosidases in the intestinal mucosal cell on the phytochemicals absorbed by enterocytes [43]. Betacyanins glycosides may be transferred to the gastrointestinal tract mucosal cells and subsequently hydrolyzed by cytosolic enzymes. It is also possible that the betalains aglycans reach the large intestine where they can be hydrolysed by β-glucosidases produced by the bacteria present in the colon but also by the alkaline environment [44]. The biologically active compounds in vitro digestibility in the gastrointestinal tract (Figure 5a–d) is a complex process, with a major impact on their release, distribution and bioavailability. In the simulated gastric juice, for the FDP variant, a decrease of the betalains content was observed, with a percentage of 35–40% after 30 min and around 40–45% after 120 min. In the simulated intestinal juice there was an increase in the release of the biologically active compounds with values between 3–3.7 times higher after 30 min of intestinal digestion and about 4–5 times higher after 120 min for all the studied products, thus suggesting a controlled release of these antioxidant compounds.

## 4. Conclusions

The fresh, dried, or freeze-dried red beet samples, enriched with *Lb plantarum* BL3, showed a high antioxidant activity, an increased content of betalains and polyphenols, so their use as nutraceuticals is clearly justified. The obtained functional food products, based on beetroot enriched with lactic acid bacteria, were obtained for the daily consumption as dietary bioactive metabolites products that could provide a high content of bioactive molecules, mainly due to the fact that these antioxidant molecules showed great potential in scavenging free radicals, compounds that damage the healthy cells hence causing many diseases. The functional products designed as ready-to-eat single dosage (100 g of fresh or dried chips variants and 10 g of freeze-dried powder) can ensure a daily intake of 10^8^–10^9^ probiotic microorganisms, a concentration that is sufficient to achieve a probiotic action on the human organism. To the best of our knowledge, this is the first time when such food products were achieved, the study being of special interest both to the scientific community and to the manufacturers from the food or pharmaceutical industry.

## Figures and Tables

**Table 1:** Texture Profile Analysis of beetroot dried chips enriched with *Lb. plantarum* BL3.

Texture Parameter	7 Days	14 Days	21 Days
Hardness, N	0.73 ± 0.008	0.75 ± 0.008	0.77 ± 0.008
Springiness, mm	1.57 ± 0.005	1.56 ± 0.008	1.52 ± 0.005

**Table 2:** Phytochemical features of the F, DC, and FDP experimental variants—The experimental variants were compared to the control (fresh beetroot cubes without LAB).

Variants	BC (mg L^−1^)	BX (mg L^−1^)	TPC (mg Gallic Acid L^−1^)	Antioxidant Activity (%)
Control	147.90 ± 5.204	64.68 ± 1.004	225.70 ± 0.034	20.19
F	226.18 ± 2.002	185.12 ± 1.229	418.30 ± 0.045	22.13
DC	349.25 ± 1.082	298.38 ± 5.854	635.80 ± 0.005	33.51
FDP	689.79 ± 4.321	786.69 ± 5.625	1314.70 ± 0.025	56.85

Note: F—fresh beetroot cubes; DC—dried beetroot chips; FDP—freeze-dried beetroot powder; BC—betacyanin, BX—betaxanthin, TPC—total polyphenols content.

**Table 3:** The drying method effect on the color of probiotic enriched beetroot samples.

	L	a	b	x	BI	a/b	b/a	Hue Value (tg^−^)
F	35.64 ± 0.34	62.76 ± 0.23	19.58 ± 0.40	0.485 ± 0.18	114.117 ± 0.32	3.20	0.3119	17.32°
DC	32.83 ± 0.18	45.25 ± 0.50	16.51 ± 0.40	0.455 ± 0.26	85.58 ± 0.31	2.70	0.3907	21.34°
FDP	34.63 ± 0.32	57.61 ± 0.49	18.73 ± 0.10	0.477 ± 0.17	98.235 ± 0.21	3.07	0.3251	18.00°
